# Low-Density Lipoprotein Receptor Apolipoprotein B Gene Polymorphism in Kurdish Patients With Severe Hypercholesterolemia

**DOI:** 10.7759/cureus.70387

**Published:** 2024-09-28

**Authors:** Saeed Sabri, Sherwan Salih, Dhia Al-Timimi

**Affiliations:** 1 Medical Chemistry, College of Medicine, University of Duhok, Duhok, IRQ

**Keywords:** apolipoprotein b, genotyping, hypercholesterolemia, kurdish population, low-density lipoprotein receptor

## Abstract

Background

Polymorphisms in the low-density lipoprotein receptor (LDLR) and apolipoprotein B 100 (APOB-100) genes have been linked to severe hypercholesterolemia in several populations. This study investigated the frequency of LDLR-Ava II and APOB-Xba I polymorphisms among Kurdish patients with severe hypercholesterolemia.

Methodology

We investigated LDLR-Ava II and APOB-Xba I gene polymorphisms in Kurdish patients attending the Duhok Specialized Laboratory Center in Duhok, Kurdistan Region, Iraq. We included a total of 80 subjects in this study, of which 40 (20 males and 20 females) had severe hypercholesterolemia, and 40 apparently healthy volunteers (21 males and 19 females) had normocholesterolemia, served as a control group. We used the polymerase chain reaction-restriction fragment length polymorphism (PCR-RFLP) technique to determine the polymorphisms of the LDLR-Ava II and APOB-Xba I genes.

Results

In those with severe hypercholesterolemia, the observed allele frequencies were AA LDLR-Ava II polymorphism (eight, 20%) followed by TT APOB-Xba I polymorphisms (six, 15%), whereas these frequencies were five (12.5%) and one (2.5%) in those with normocholesterolemia, respectively. The AA genotype group had considerably higher cholesterol and LDL-C levels compared with the GG genotype group. A similar pattern was observed when comparing the TT and CC genotype groups.

Conclusions

Our results showed a high frequency of AA LDLR-Ava II polymorphism in conjunction with TT APOB-Xba I polymorphism which may be strongly associated with hypercholesterolemia in the Kurdish population.

## Introduction

The high incidence of hypercholesterolemia is a matter of great concern, as the concomitantly elevated levels of low-density lipoprotein cholesterol (LDL-C) pose a major risk factor for the development of cardiovascular diseases (CVD) [1]. Hypercholesterolemia (HC) can be either due to primary (genetic or familial) or secondary (acquired) causes. Genetic mutations of the LDL receptor (LDLR) gene account for 85% of familial causes. Other factors include defective apolipoprotein B (APOB), proprotein convertase subtilisin/kexin type 9 (PCSK9) gene gain-of-function mutations, low-density lipoprotein receptor adaptor protein (LDLRAP1) mutation, and polygenic HC [2, 3].

Familial hypercholesterolemia (FH) is a gene-related metabolic disorder, and it is mainly caused by pathogenic mutations in LDLR, APOB, PCSK9, and LDLRAP1 affecting the LDL receptor (LDLR) pathway [4, 5]. Mutational screening of these genes may play a role in the diagnosis of FH and identification of the affected relatives early [6]. Variants in LDLR have been estimated to account for more than 85% of all FH-causing mutations [7]. The LDLR gene is located on the short arm of chromosome 19 (19p13.2), with a length of approximately 45 kb, and comprises 18 exons and 17 introns. A common Ava II (rs5925, c.1959 T > C, change of codon GTT → GTC; p.Val653Val) polymorphic site present in exon 13 has been found to be associated with variation in serum lipid levels. Single nucleotide variants (SNVs) represent 65% of all LDLR variants and are mainly missense (40-50%), frameshift (15-20%), nonsense (12-15%), and splicing mutations (8-10%) [7, 8].

The human APOB gene is located on the short arm of chromosome 2 (2p23-24) with a length of approximately 43 kb and contains 29 exons and 28 introns. The cleavage site for Xba I is located within exon 26 of the APOB gene and arises due to a single base variation (replacing a cytosine (C) with thymine (T) at nucleotide 7673); this substitution results in a change from the codon ACC → ACT at 2488th position. Variants in the APOB gene leading to FH are those causing an impaired binding of APOB to LDLR [9], i.e., mainly those present in the gene region coding for the LDLR binding domains (exons 26 and 29), even though variants present in other regions were described [10]. The APOB gene contains over 5000 polymorphic sites; several sites are described as modifiers of cardiovascular risk [11]. One of the most studied APOB polymorphisms is Xba I polymorphism (rs693 or C7673T). It is silent as there is no amino acid change at codon 2488 of the genome but it is associated with variations in plasma lipid levels [12]. However, little is known about the LDLR and APOB gene polymorphisms in the Kurdish population, especially among those with severe hypercholesterolemia. As severe hypercholesterolemia is an established risk factor of CVD, it is important to investigate the distribution of LDLR and APOB gene polymorphisms in the Kurdish population, as this polymorphism might provide insight into the nature of the disease. Therefore, in this study, we investigated the frequency of LDLR-Ava II and APOB-Xba I polymorphisms in patients with severe hypercholesterolemia not suffering from any secondary cause, as well as a group of normocholesterolemia subjects.

## Materials and methods

 Study population

We obtained a representative sample using a random sampling procedure for patients with severe hypercholesterolemia attending the Duhok Specialized Laboratory Center, referred from the Endocrinology Department at the Azadi Teaching Hospital in Duhok, Kurdistan Region, Iraq, between January 2021 and March 2022. We registered a total of 40 patients with severe hypercholesterolemia with prominent signs and symptoms of CVD (TC >290 mg/dL) and 40 apparently healthy volunteers as the control group for the study over six consecutive months, ranging in age from 3 to 65 years. The inclusion criteria were patients with severe hypercholesterolemia who were not suffering from any secondary cause (such as diabetes mellitus, hypothyroidism, nephrotic syndrome, chronic renal disease, chronic obstructive liver disease, and other causes of secondary hypercholesterolemia), did not drink alcohol, and had a positive family history of CVD. The control group comprised 40 apparently healthy volunteers ranging in age from 9 to 55 years, with normal serum TC levels (TC <200 mg/dL). After we interviewed the participants, we explained the purpose of the study to each of them and received verbal consent.

Following an overnight fast, 5 mL of whole blood was collected from each participant through venipuncture. The blood was then divided into three parts: the first part (2 mL) was collected in a test tube containing ethylene diamine tetra-acetic acid (EDTA) and held at −50 °C until it was used for the analysis of LDLR-Ava II and APOB-Xba I gene polymorphism by polymerase chain reaction-restriction fragment length polymorphism (PCR-RFLP); the second part (1 mL) was placed in a test tube containing EDTA for glycated hemoglobin (HbA1c) testing; and the third part (2 mL) was placed in an empty gel test tube for serum. The Cobas 6000 (open, automated, discrete, and random access; Roche, Mannheim, Germany) clinical chemistry analyzer was used to determine serum concentrations of TC, LDL-C, TG, HDL-C, glucose, HbA1c, C-reactive protein (CRP) and thyroid- stimulating hormone (TSH).

Analysis of gene polymorphism

Genomic DNA purification was done at the Duhok Specialized Laboratory Center. A purification kit was used for genomic DNA isolation from frozen whole blood cells and placed in a tube with EDTA according to the manufacturer's recommended protocols (Add-Bio Genomic DNA Extraction Kit, Korea). The quantity and quality of the genomic DNA samples were measured using a nanophotometer (Thermo Scientific Nanodrop 2000 Spectrophotometer, USA) before storing them at −20 °C for further use.

**Figure 1:**
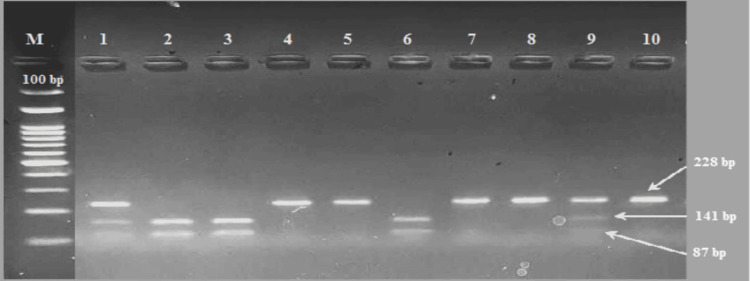
Electrophoresis of agarose gel (2.5%) for LDLR-Ava II polymorphism M - 100 bp DNA Marker (ladder) lane indicating molecular weight; LDLR - low-density lipoprotein receptor Lanes 2, 3, and 6 correspond to AA polymorphism. Lanes 1 and 9 correspond to GA polymorphism. Lanes 4, 5, 7, 8, and 10 correspond to GG polymorphism.

Genotyping of the LDLR-Ava II polymorphism was performed using the PCR-RFLP technique. PCR amplification was carried out with the forward primer 5'-GTCATCTTCCTTGCTGCCTGTTTAG-3' and the reverse primer 5'-GTTTCCACAAGGAGGTTTCAAGGTT-3'. After initial denaturation at 94°C for three minutes, the reaction mixture was subjected to 30 cycles of 30s denaturation at 94°C, 30s annealing at 55°C and extension of 60 seconds at 72°C, followed by a final five-minute extension at 72 °C. After digestion of the amplified DNA by the restriction enzyme Ava II (New England Biolabs Inc.R0153S), the genotypes were identified by electrophoresis on 2.5% agarose gels and visualized with red dye staining ultraviolet illumination. After digestion, three genotypes were detected: AA homozygous mutation, with restriction site present (141 + 87 bp), GA heterozygous mutation with restriction site present (228 + 141 + 87 bp), and GG (228 bp) homozygous wild genotype without the restriction site [13] (Figure 1).

**Figure 2:**
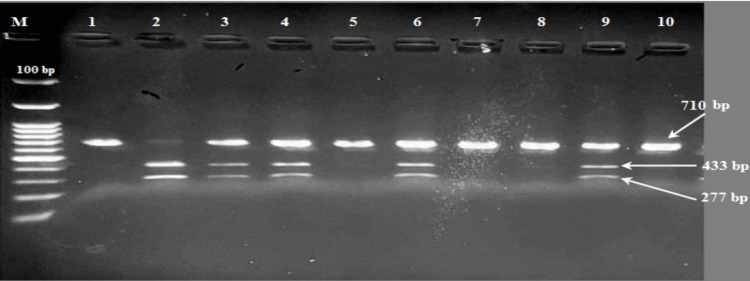
Electrophoresis of agarose gel (2.5%) for APOB-Xba I polymorphism M - 100 bp DNA marker (ladder) lane indicating molecular weight Lanes 2 corresponds to TT polymorphism. Lanes 3, 4, 6, and 9 correspond to GA polymorphism. Lanes 1, 5, 7, 8, and 10 correspond to GG polymorphism.

Genotyping of APOB-Xba I polymorphism was performed by PCR-RFLP. PCR amplification was carried out with the forward primer 5' GGAGACTATTCAGAAGCTAA-3' and the reverse primer 5'GAAGAGCCTGAAGACTGA-3'. After initial denaturation at 94°C for three minutes, the reaction mixture was subjected to 30 cycles of 30s denaturation at 94°C, 30s annealing at 55°C and extension 60s at 72°C, followed by a final five-minute extension at 72 °C. After the digestion of the amplified DNA by the restriction enzyme Xba I (New England Biolabs Inc.R0145S), the genotypes were identified by electrophoresis on 2.5% agarose gels and visualized with red dye staining ultraviolet illumination. After digestion, three genotypes were detected: TT homozygous mutation, with restriction site present (433+ 277 bp), CT heterozygous mutation with restriction site present (710 + 433+ 277 bp), and CC (710 bp) homozygous wild genotype without the restriction site [13] (Figure 2).

Ethical approval

We obtained ethical approval for the study from the General Directorate of Health in Duhok Governorate, with document number 13072021-7-15 (including the number and the date, July 13, 2021). We also obtained informed consent from the respondents after explaining the objective of the study.

Statistical analysis

We used SPSS Statistics for Windows, Version 25.0 (IBM Inc., Armonk, New York) to analyze the data. The laboratory parameter values are displayed as a mean and standard deviation (SD). We used the chi-square test to compare variables, and the Student's independent t-test to compare variables between groups. A p-value of ≤0.05 was considered statistically significant. To ascertain the relationship between genotypes, odds ratios (ORs) and related 95% confidence intervals (CIs) were used.

## Results

Table 1 shows the general characteristics of the participants. We found significant differences in serum TC (p<0.001), TG (p=0.006), LDL-C (p<0.001), and CRP level (p=0.007) between the normocholesterolemia and hypercholesterolemia groups. We found no significant differences with respect to age, body mass index, glucose, HbA1c, and TSH.

**Table 1:** General characteristics of the study participants The data are represented as mean ± SD. Student's independent t-test was used to compare the variables between normocholesterolemia and hypercholesterolemia groups. A p-value of ≤0.05 was considered statistically significant (*). N - number; SD - standard deviation; BMI - body mass index; WC - waist circumference; SBP - systolic blood pressure; DBP - diastolic blood pressure; TC - total cholesterol; TG - triglyceride; LDL-C - low-density lipoprotein cholesterol; HDL-C - high-density lipoprotein cholesterol; HbA1c - hemoglobin A1c; TSH - thyroid-stimulating hormone; CRP - C-reactive protein

Characteristics	Subjects with normocholesterolemia (N=40) mean±SD	Patients with hypercholesterolemia (N=40) mean±SD	p-value
Age (years)	33.5±10.2	34.9±17.58	0.689
BMI (kg/m2)	24.0±3.8	25.0±4.5	0.291
WC (cm)	87.2±8.6	84.3±16.7	0.327
SBP (mmHg)	113.7±14.0	122.5±15.9	0.015^*^
DBP (mmHg)	72.7±10.3	77.5±10.3	0.049*
TC (mg/dL)	157.1±25.2	363.5±137.2	< 0.001^*^
TG (mg/dL)	119.1±66.6	174.1±98.9	0.006^*^
LDL-C (mg/dL)	89.6±21.4	278.6±126.5	< 0.001^*^
HDL-C (mg/dL)	47.6±9.1	48.2±8.0	0.066
Glucose (mg/dL)	92. 6±8.0	93.8±12.2	0.102
HbA1c (%)	4.7±0.4	5.6±1.3	0.061
TSH (µIU/mL)	2.0±1.5	2.3±1.0	0.299
CRP (mg/L)	2.0±2.2	4.3±4.7	0.007^*^

Table 2 shows the genotype and allele frequencies of the LDLR-Ava II and APOB-Xba I gene polymorphisms. In the case of polymorphism of the LDLR gene, the AA genotype denotes homozygotes with two alleles in which the restriction site is present; the GA genotype denotes heterozygotes with one allele with the site present and one allele with the site absent, and the GG genotype denotes homozygotes without the restriction site. The GG genotype was observed to be the most common allele set found in patients with severe hypercholesterolemia as compared to the normocholesterolemia group.

**Table 2:** Frequency distribution of LDLR-Ava II and APOB-Xba I polymorphisms among study groups Data are represented as N (%). The Chi-squared test (χ2) was used to compare the frequency distribution of genotypes and alleles of LDLR-Ava II and APOB-Xba I genes. A p-value of ≤0.05 was considered statistically significant (*). N - number; OR - odds ratio; LDLR - low-density lipoprotein receptor; APOB - apolipoprotein B

Study group	Ava II genotypes, N (%)			Xba I genotypes, N (%)			LDLR-Ava ll / APOB-Xba I allele frequency (%)			
	GG	GA	AA	CC	CT	TT	G	A	C	T
Patients with hypercholesterolemia (N=40)	16 (40.0)	16 (40.0)	8 (20.0)	13 (32.5)	21 (52.5)	6 (15.0)	0.60	0.40	0.58	0.42
Subjects with normocholesterolemia (N=40)	11 (27.5)	24 (60.0)	5 (12.5)	18 (45.0)	21 (52.5)	1 (2.5)	0.57	0.43	0.72	0.28
OR	1.75	0.44	1.75	0.58	1.0	6.8	-	-	-	-
p-value	0.237	0.073	0.363	0.252	1.000	0.047*	-	-	-	-

According to the polymorphism distribution analysis, patients with severe hypercholesterolemia had higher frequencies of AA genotype than those with normocholesterolemia (20.0% vs. 12.5%, respectively). A similar pattern was observed for the TT polymorphism, where the TT genotype denoted homozygosity for two alleles in which the restriction site is present, whereas the CC genotype denoted homozygosity for the existence of a naturally occurring APOB-Xba I without the restriction site. The CT genotype denotes heterozygosity with one allele with the site present and one allele with the site absent. The distribution of APOB-Xba I showed that the hypercholesterolemia group had a higher frequency of the TT genotype compared to the normocholesterolemia group (15% vs. 2.5%), and the difference was statistically significant (OR=6.8, p=0.047).

To determine which of the alleles was significantly associated with serum TC and LDL-C levels, we used the Student's t-test to compare the means between groups, as shown in Table 3; we found a significant difference in TC and LDL-C between those with AA and GG alleles (p<0.001). Regarding APOB-Xba I, the TT group had higher TC and LDL-C levels compared to the CC group, but the difference did not show statistical significance (p=0.219 and p=0.279, respectively).

**Table 3:** Total cholesterol and LDL-C levels stratified by Ava II and Xba I polymorphisms in patients with severe hypercholesterolemia. Data are represented as mean ± SD. Student's independent t-test was used to compare the TC and LDL-C values between the different genotypes of LDLR-Ava II and APOB-Xba I genes in patients with severe hypercholesterolemia. A p-value of ≤0.05 was considered statistically significant (*). SD - standard deviation; TC - total cholesterol; LDL-C - low-density lipoprotein cholesterol; LDLR - low-density lipoprotein receptor; APOB - apolipoprotein B

Genotype	TC (mg/dL) mean ± SD	LDL-C (mg/dL) mean ± SD
Ava II		
GG	289.8±39.3	208.8±29.6
AA	495.5±213.8	405.8±199.9
p-value	< 0.001*	< 0.001*
AA	495.5±213.8	405.8±199.9
GA	371.3±104.5	284.6±90.5
p-value	0.066	0.051
Xba I		
CT	368.0±143.2	290.3±143.6
TT	415.3±214.9	305.6±168.9
p-value	0.529	0.826
CC	332.3±72.9	274.1±63.4
TT	415.3±214.9	305.6±168.9
p-value	0.219	0.279

Table 4 compares the ORs of the LDLR-Ava II and APOB-Xba I polymorphism genotypes in all participants. The OR of AA versus GG (0.38) was higher than that observed in AA versus GA (0.194). Regarding APOB-Xba I a higher OR was observed for TT versus CC (0.151) compared to TT versus CT (0.086). A higher OR was found in AA and GA versus GG (3.853) compared to the OR of TT and CT versus CC (2.490).

**Table 4:** Comparison of LDLR-Ava II and APOB-Xba I polymorphism genotypes in all participants Data are represented as odds ratio and 95% CI. Odds ratio and 95% confidence intervals were used to compare LDLR-Ava II and APOB-Xba I genotypes in all participants. A p-value of ≤0.05 was considered statistically significant (*). OR - odds ratio; 95% CI - 95 percentile confidence interval; LDLR - low-density lipoprotein receptor; APOB - apolipoprotein B

Genotypes	OR	95% Cl	*P*-value
LDLR-Ava II			
AA vs. GA	0.194	0.09-0.40	0.232
AA vs. .GG	0.380	0.17-0.80	0.131
AA and GA vs. GG	3.853	2.00-7.42	< 0.001*
APOB-Xba I			
TT vs. CT	0.086	0.03-0.21	0.237
TT vs.CC	0.151	0.06-0.37	0.234
TT and CT vs. CC	2.490	1.32-4.71	0.004*

Table 5 presents the relationship between TC and LDL-C levels and both LDLR-Ava II and APOB-Xba I polymorphisms in all participants. For LDLR-Ava II, the mean serum TC and LDL-C levels were significantly higher in the AA polymorphism group than in the GG group (p<0.001). For APOB-Xba I, TC and LDL-C levels were significantly higher in the TT polymorphism group compared to the CC polymorphism group (p=0.012 and p=0.016, respectively).

**Table 5:** Total cholesterol and LDL-C levels stratified by LDLR-Ava II and APOB-Xba I polymorphisms in all participants. Data are represented as mean ± SD. A p-value of ≤0.05 was considered statistically significant (*). Student's independent t-test was used to compare the TC and LDL-C values between the different genotypes of LDLR-Ava II and APOB-Xba I genes in all participants. SD - standard deviation; TC - total cholesterol; LDL-C - low-density lipoprotein cholesterol; LDLR - low-density lipoprotein receptor; APOB - apolipoprotein B

Genotype	TC (mg/dL) mean ± SD	p-value	LDL-C (mg/dL) mean ± SD	p-value
LDLR-Ava II				
GG	210.8±71.7	< 0.001*	137.7±63.5	< 0.001*
AA	364.6±238.2		284.3±221.8	
GA	283.4±135.2	0.176	204.5±120.9	0.148
AA	364.6±238.2		284.3±221.8	
GA	283.4±135.2	0.006^*^	204.5±120.9	0.004*
GG	210.8±71.7		137.7±63.5	
APOB-Xba I				
CT	260.4±148.9	0.086	187.1±145.6	0.167
TT	375.1±223.1		273.1±176.6	
CC	234.2±98.3	0.012*	159.8+86.6	0.016*
TT	375.1±223.1		273.1±176.6	
CT	260.4±148.9	0.398	187.1±145.6	0.355
CC	234.2±98.3		159.8+86.6	

## Discussion

To our knowledge, this is the first prospective investigation that was done on genotypes and the association between LDLR-Ava II / APOB-Xba I gene polymorphisms and hypercholesterolemia in the Kurdish population in Duhok province, Kurdistan Region, Iraq. The data for this study were derived from a cross-sectional survey centered on lipid disorders and related risk factors (Duhok Lipid Disorders Study, DLDS). The DLDS was conducted in Duhok City from January 2021 to December 2022, aiming to investigate the prevalence and genetic factors for lipid disorders [14]. Our results showed higher genotypic and allelic frequencies of the AA genotype between study groups, and a similar pattern was observed with a significant difference for the TT genotype. The results confirmed an association between AA polymorphism and hypercholesterolemia, as the OR of AA vs. GG was about two times higher than that observed in AA vs. GA. These results are in accordance with previous studies [15, 16]. Moreover, we reported the most frequent allele identified in patients with severe hypercholesterolemia was LDLR-Ava II polymorphism AA compared to that in subjects with normocholesterolemia, indicating that LDLR-Ava II polymorphism AA is a genetic marker of susceptibility to severe hypercholesterolemia in Kurdish population.

Importantly, a strong association between LDLR-Ava II polymorphism AA and cholesterol values has been reported in several populations [17, 18]. The study by Onwe et al. also reported a significant association between cholesterol levels and the LDLR-Ava II gene polymorphism [19]. Zihlif et al. observed that the highest serum cholesterol concentrations were among individuals with a high frequency of LDLR-Ava II [20]. A study by Starcevic et al. supported this observation since it reported that cholesterol was strongly associated with LDLR gene polymorphism even after adjustment for potential risk factors [21]. According to our statistical analysis, we found that LDLR-Ava II polymorphism AA was the most powerful genetic marker of susceptibility to severe hypercholesterolemia, which is similar to the results of other studies [22, 23].

Regarding APOB-Xba I, a higher OR was observed for TT vs. CC compared to TT vs. CT. Nevertheless, we observed a lower OR for TT and CT vs. CC compared to AA and GA vs. GG, indicating that APOB-Xba I gene polymorphism is a less significant genetic marker compared to LDLR-Ava II polymorphism, as reported elsewhere [17]. However, recent evidence supports the association of APOB-Xba I gene polymorphism with CVD risk and LDL-C levels in several populations [11]. These results were contrary to a previous study that suggested the T allele may have a beneficial effect on the level of serum cholesterol [24]. According to a study conducted in the western region of Saudi Arabia, coronary artery disease patients with APOB-Xba I genotypes had significantly higher TC, LDL-C, and triglyceride values than controls [25]. Studies on the link between LDLR-Ava II and/ or APOB-Xba I genotypes and hypercholesterolemia in several populations have yielded controversial results [26]; some reported a significant association between both gene polymorphisms and hypercholesterolemia [13], whereas others reported no such association [27]. Furthermore, several studies have recognized the connection between LDLR-Ava II and APOB-Xba I polymorphisms and elevated serum levels of TC and LDL-C [13, 28].

Limitations

This study has certain limitations. The data obtained from subjects living in Duhok City may not represent all Kurds living in other parts of the Kurdistan region. Furthermore, identification of the PCSK9 and LDLRAP1 genes was not conducted. Despite that, this study demonstrated an association between LDLR-Ava II polymorphism AA and APOB-Xba I polymorphism TT with hypercholesterolemia in the Kurdish population.

## Conclusions

In conclusion, our study showed that Kurdish patients with severe hypercholesterolemia had higher frequencies of LDLR-Ava II polymorphism AA and APOB-Xba I polymorphism TT than those with normocholesterolemia, which may be hereditary indicators of vulnerability to severe hypercholesterolemia in Kurdish population. Doing a large genetic study, especially on APOB-Xba I polymorphism TT as a genetic marker of susceptibility to severe hypercholesterolemia, may be of great importance. Furthermore, mutational screening of these genes is recommended to confirm the diagnosis of FH and identify affected relatives early.
